# Economic Burden and Healthcare Trajectories of Patients Awaiting Heart Transplantation in a French Tertiary Center

**DOI:** 10.3389/ti.2025.13703

**Published:** 2025-03-04

**Authors:** Jamal Atfeh, Pascale Guerre, Laurent Sebbag, Matteo Pozzi, Laure Huot

**Affiliations:** ^1^ Hospices Civils de Lyon, Pôle de Santé Publique, Service d’Evaluation Economique en Santé, Lyon, France; ^2^ Université Claude Bernard Lyon 1, Research on Healthcare Performance (RESHAPE), INSERM U1290, Lyon, France; ^3^ Université Claude Bernard Lyon 1, Health Systemic Process, EA 4129 Research Unit, Lyon, France; ^4^ Hospices Civils de Lyon, Hôpital Louis Pradel, Service Insuffisance Cardiaque Assistance et Transplantation, Lyon, France; ^5^ Hospices Civils de Lyon, Hôpital Louis Pradel, Service de Chirurgie Cardiaque, Lyon, France

**Keywords:** health economics, heart transplantation, pathway, waiting list, donor pool

## Abstract

Heart transplantation (HT) is the gold standard treatment of end-stage heart failure, but organ shortage remains a challenge. This retrospective cohort study assesses the economic burden and healthcare pathways of patients awaiting HT in a French tertiary center. Direct healthcare resources were collected and valued, and a state sequence analysis was performed. Ninety-two adult patients were included, with 67 (73%) undergoing HT within a median waiting time of 2 months. The mean cost per patient was €21,324.05 with an average of 2.71 hospitalizations. Four clusters were identified. Type 1 patients (n = 43) underwent HT within 1 month, with a mean cost of €5,820.12 per patient. Only 4 (25%) Type 2 patients (n = 16) underwent HT within 30 months, as they were not prioritized for HT, with a mean cost of €22,285.32 per patient. Type 3 patients (n = 20) underwent HT within 10 months, but incurred higher costs (€27,541.11) compared to Type 2 patients over a shorter period. Despite high transplant priority, Type 4 patients (n = 13) died before HT within 3 months, with a mean cost of €61,858.45 and 3 hospitalizations. This work highlights the economic burden of organ shortage. The use of novel heart preservation devices (such as *ex-vivo* perfusion systems) could help to expand the donor pool and alleviate this burden, but these aspects need to be further investigated.

## Introduction

Heart transplantation is still the gold standard for carefully selected patients with end-stage heart failure refractory to guidelines-directed optimal medical treatment, with a reported median survival of 12.5 years [1–3]. Moreover, one-year survival on the heart transplantation waiting list has increased up to 67.8% in the 2011–2017 period due to improvements in the management of these severe patients [4]. Nevertheless, one of the key challenges worldwide is to overcome the large imbalance between organ supply and demand for heart transplantation [5]. In France in 2019 before the pandemic coronavirus disease, 573 patients were scheduled on the heart transplantation waiting list but only 425 underwent cardiac transplantation during the same year due to a shortage of available donors [6].

Data on the costs associated with the medical management (apart surgery) of patients with end-stage heart failure listed for heart transplantation are lacking. These data are important because they highlight the economic burden of organ shortage and the potential of strategies to expand the donor pool to help alleviate this burden, such as using *ex vivo* perfusion systems [7]. In a context of limited healthcare resources, our objective was to evaluate the economic burden of patients awaiting heart transplantation in a French tertiary center. A cost of illness (COI) study was conducted alongside a state sequence analysis to compare the economic outcomes with patients’ healthcare trajectories.

## Materials and Methods

### Study Design, Setting and Population

A retrospective cohort study was conducted in accordance with the provisions of the French Law and the European General Data Protection Regulation. The study was registered on the National Data Protection Commission register authorized for the Lyon University Hospital (n°22-5946) and has received a favorable opinion from our ethics and scientific committee on 21 December 2022 (n°22-946). All eligible patients were informed and could object to the use of their data.

We included adult patients (aged 18 or older) who were newly scheduled on our heart transplantation waiting list between 1 January 2018 and 31 December 2020. January 2018 was chosen because a new heart allocation system was introduced in France at that time [8]. Participants awaiting multi-organ transplantations were excluded. The main outcome was access to heart transplantation. The cohort entry date was the date of registration on the waiting list. The cohort exit date was the date of heart transplantation surgery, death or the end of the study period (30 June 2022), whichever came first. Patients lost to follow-up would be considered non-transplanted (worst-case scenario).

### Data Collection

Data were collected through computerized medical records at individual level for all participants. Baseline patient clinical characteristics were collected at the time of registration on the heart transplantation waiting list: age, body mass index (BMI), New York Heart Association (NYHA) functional classification, indication for heart transplantation, Cardiac Risk Index (CRI) (i.e., a one-year waitlist mortality predictive score based on candidate characteristics, and part of the 2018 French heart allocation system [9]), mechanical circulatory support (temporary or durable), inotropic support, medical history, comorbidities and risk factors. Direct healthcare resource consumptions (i.e., hospitalizations, outpatient medical consultations and outpatient medical procedures) were also collected.

### State Sequence Analysis

State Sequence Analysis is an epidemiological method derived from social sciences which can be used to describe and characterize typologies of longitudinal sequences such as healthcare trajectories [10–12]. Herein, six states were predefined: hospitalization, medical procedure, medical consultation, heart transplantation, death, waiting list. Once a patient experienced heart transplantation or death, he would remain in this state (irreversible states). The distance between each pair of patient sequences was then measured using Optimal Matching, a commonly used dissimilarity measure method with an insertion/deletion cost of 1 and a substitution cost matrix estimated based on observed transition rates between states [13]. Agglomerative hierarchical clustering using Ward’s criterion on the dissimilarity matrix was then performed to create homogeneous clusters of patients and optimal number of clusters was chosen using the inertia curve [11].

### Economic Evaluation

COI studies are designed not only to evaluate the costs attributable to the treatment of a particular illness but also to estimate actual illness-related costs [14]. The economic evaluation was conducted from the healthcare system perspective, which focuses solely on healthcare production and accounts for all monetary costs of healthcare, regardless of who bears the cost [15, 16]. Time horizon was set from the cohort entry to cohort exit dates. Given that our goal was to assess the economic burden of patients on the heart transplantation waiting list (not to compare any interventions at different points in time), we chose not to discount costs regardless of patients awaiting for more than 12 months. This methodological choice was consistent with our objective to estimate the actual expenses involved to manage these patients. All costs were expressed in euros (€) at 2023 price year and adjusted for inflation based on the French National Institute of Statistics and Economic Studies (INSEE) Consumer Price Indices of the healthcare products and services [17].

A top-down micro-costing approach was taken [18]. After identification, hospital stays were classified per Diagnosis Related Group (DRG) using the local Medicine-Surgery-Obstetrics Medical IT system (PMSI). Hospital stays were then valued using the French National Cost Study (NCS), a study based on the cost-accounting of a sample of public and private French institutions, which produces the closest valuation to the hospital production cost [15]. The average cost of stay excluded structural costs as well as cost of products (medicines and medical devices) funded on top of Healthcare Resource Group (HRG) based tariffs, which were additionally valued on the basis of their reference price stated in the French Official Gazette. Outpatient medical consultations and outpatient medical procedures were respectively valued on the basis of reimbursement tariffs of the French National Health Insurance and the French Joint Classification of Medical Procedures (CCAM).

In order to respect the cohort entry and exit dates and to properly exclude heart transplantation-related costs from the evaluation, we performed a specific valuation methodology on certain hospital stays (Supplementary Figure S1). When the date of enrolment on the waiting list occurred during a given hospital stay, the DRG provided by the Medical IT system was valued using the NCS and divided by its mean national length of stay (also provided by the NCS) to obtain a mean hospital cost per day. It was then multiplied by the actual patient’s length of stay between the date of enrolment and the date of hospital discharge. When the date of enrolment on the waiting list and the date of heart transplantation surgery occurred on the same hospital stay, a standardized DRG of cardiac decompensation (05M093) was applied instead of the heart transplantation Medical IT system DRG and patient’s length of stay between the date of enrolment and the date of heart transplantation was taken into account. The same standardized DRG was applied when a given hospital admission led to transplantation (i.e., heart transplantation was not the hospitalization reason) but patient’s length of stay from admission to heart transplantation was taken into account.

### Statistical Analysis

Descriptive quantitative data were presented using medians and first and third quartiles. Descriptive qualitative data were presented using integer numbers and percentage frequencies. Homogeneous clusters of patients obtained from the state sequence analysis were described according to patient baseline characteristics and to their healthcare resource consumption. The status at the cohort exit date (transplanted, dead, non-transplanted) and the time from heart transplantation list registration to heart transplantation (or death) were also presented per cluster. Exploratory bivariate analyses were conducted to analyze patient baseline covariates according to cluster types. A bivariate association was sought using the Chi2 test for the qualitative variables (or the Fisher’s exact test in case of insufficient conditions of performance) and using the Kruskall Wallis non parametric test for the quantitative variables. A significance threshold of 5% was set, and all tests were two-tailed.

Mean costs per patient and mean quantities per cost item were presented, assorted with their Bias-Corrected and accelerated bootstrapped (R = 10,000) 95% Confidence Intervals (CI) to assess uncertainty around our point estimates. Cost differences between groups were considered statistically significant if the bootstrapped 95% CIs did not overlap. All analyses were performed using R (version 4.2.2) within R Studio software. The R package “TraMineR” was used to perform the state sequence analysis [13].

## Results

### Baseline Patient Characteristics

During the study period, 92 patients were included (median age of 52 years, male sex 71%). Medical history, comorbidities and risk factors are also summarized in Table 1. Ischemic cardiomyopathy and dilated cardiomyopathy were the most common indications for heart transplantation (41% and 34%, respectively). The median CRI, which assesses priority for heart transplantation based on candidate characteristics was 21. Inotropic support was required before heart transplantation in 24 (26%) patients. Twenty-seven (29%) patients were bridged to heart transplantation on temporary (i.e., extracorporeal membrane oxygenation [ECMO]; n = 17, 18%) or durable (i.e., long term ventricular assist device [LVAD]; n = 10, 11%) mechanical circulatory support.

**TABLE 1:** Baseline patient characteristics.

	All patients (n = 92)	Type 1 (n = 43)	Type 2 (n = 16)	Type 3 (n = 20)	Type 4 (n = 13)	p-value^a^
Age (years), median (Q1-Q3)	52 (43–59)	53 (44–59)	47 (42–56)	52 (42–60)	58 (51–60)	
Sex, n (%)						0.045
Male	65 (71)	26 (60)	15 (94)	13 (65)	11 (85)	
Female	27 (29)	17 (40)	1 (6.3)	7 (35)	2 (15)	
BMI (kg/m^2^), median (Q1-Q3)	26.0 (23.2–29.6)	24.7 (22.0–28.1)	26.1 (25.6–28.2)	28.9 (26.0–30.3)	25.2 (23.7–28.3)	
BMI ≥30 kg/m^2^, n (%)	21 (23)	9 (21)	3 (19)	6 (30)	3 (23)	
Indication for heart transplantation, n (%)						
Ischemic cardiomyopathy	38 (41)	19 (44)	6 (38)	6 (30)	7 (54)	
Dilated cardiomyopathy	31 (34)	16 (37)	4 (25)	7 (35)	4 (31)	
Hyperthrophic cardiomyopathy	7 (7.6)	1 (2.3)	3 (19)	3 (15)	0 (0)	0.041
Valvular cardiomyopathy	1 (1.1)	0 (0)	1 (6.3)	0 (0)	0 (0)	
Adult congenital heart disease	3 (3.3)	2 (4.7)	1 (6.3)	0 (0)	0 (0)	
Graft failure	2 (2.2)	1 (2.3)	0 (0)	1 (5.0)	0 (0)	
Graft coronary heart disease	1 (1.1)	0 (0)	1 (6.3)	0 (0)	0 (0)	
Others	9 (9.8)	4 (9.3)	0 (0)	3 (15)	2 (15)	
NYHA Functional Classification, n (%)						<0.001
Class II	24 (26)	8 (19)	8 (50)	6 (30)	2 (15)	
Class III	46 (50)	22 (51)	7 (44)	14 (70)	3 (23)	
Class IV	22 (24)	13 (30)	1 (6.3)	0 (0)	8 (62)	
CRI, median (Q1-Q3)	21 (14–28)	23 (18–29)	11 (9–16)	19 (16–25)	33 (26–36)	<0.001
Temporary MCS (i.e., ECMO), n (%)	17 (18)	9 (21)	0 (0)	2 (10)	6 (46)	0.009
Durable MCS (i.e., LVAD), n (%)	10 (11)	2 (4.7)	1 (6.3)	6 (30)	1 (7.7)	0.028
Inotropic support, n (%)	24 (26)	13 (30)	0 (0)	1 (5.0)	10 (77)	<0.001
Cardiovascular risk factors, n (%)						
Hypertension	19 (21)	5 (12)	4 (25)	4 (20)	6 (46)	
Diabetes	20 (22)	9 (21)	4 (25)	3 (15)	4 (31)	
Smoking						
Active smoking	11 (12)	7 (16)	1 (6.3)	2 (10)	1 (7.7)	
Previous smoking	46 (50)	19 (44)	9 (56)	11 (55)	7 (54)	
Comorbidities, n (%)						
Chronic renal failure	13 (14)	6 (14)	0 (0)	4 (20)	3 (23)	
Arrhythmia	55 (60)	26 (60)	11 (69)	11 (55)	7 (54)	
ICD	63 (68)	28 (65)	15 (94)	13 (65)	7 (54)	
Cardiac resynchronisation therapy	24 (26)	11 (26)	4 (25)	6 (30)	3 (23)	
Familial cardiomyopathy	16 (17)	5 (12)	6 (38)	3 (15)	2 (15)	
Peripheral arterial disease	5 (5.4)	1 (2.3)	1 (6.3)	1 (5.0)	2 (15)	
Concomitant pulmonary disease	4 (4.3)	2 (4.7)	0 (0)	2 (10)	0 (0)	
Previous CVA	11 (12)	3 (7.0)	3 (19)	4 (20)	1 (7.7)	
History of cancer	9 (9.8)	4 (9.3)	0 (0)	4 (20)	1 (7.7)	
Previous cardiac surgery	18 (20)	11 (26)	2 (13)	3 (15)	2 (15)	
Previous thoracic surgery	1 (1.1)	0 (0)	0 (0)	0 (0)	1 (7.7)	
Venous thromboembolic disease	4 (4.3)	3 (7.0)	0 (0)	1 (5.0)	0 (0)	

BMI, Body Mass Index; CRI, Cardiac Risk Index; CVA, Cerebrovascular Accident; MCS, Mechanical Circulatory Support; ECMO, Extracorporal Membrane Oxygenation; LVAD, Long term Ventricular Assist Device; ICD, Implantable Cardioverter Defibrillator; NYHA, New York Heart Association.

^a^ Chi2 test or Fisher’s exact test for qualitative variables; Kruskall Wallis test for quantitative variables. A significance threshold of 5% was set, and all tests were two-tailed. For clarity, only statistically significant p-values are shown.

### Description of Clusters

**FIGURE 1:**
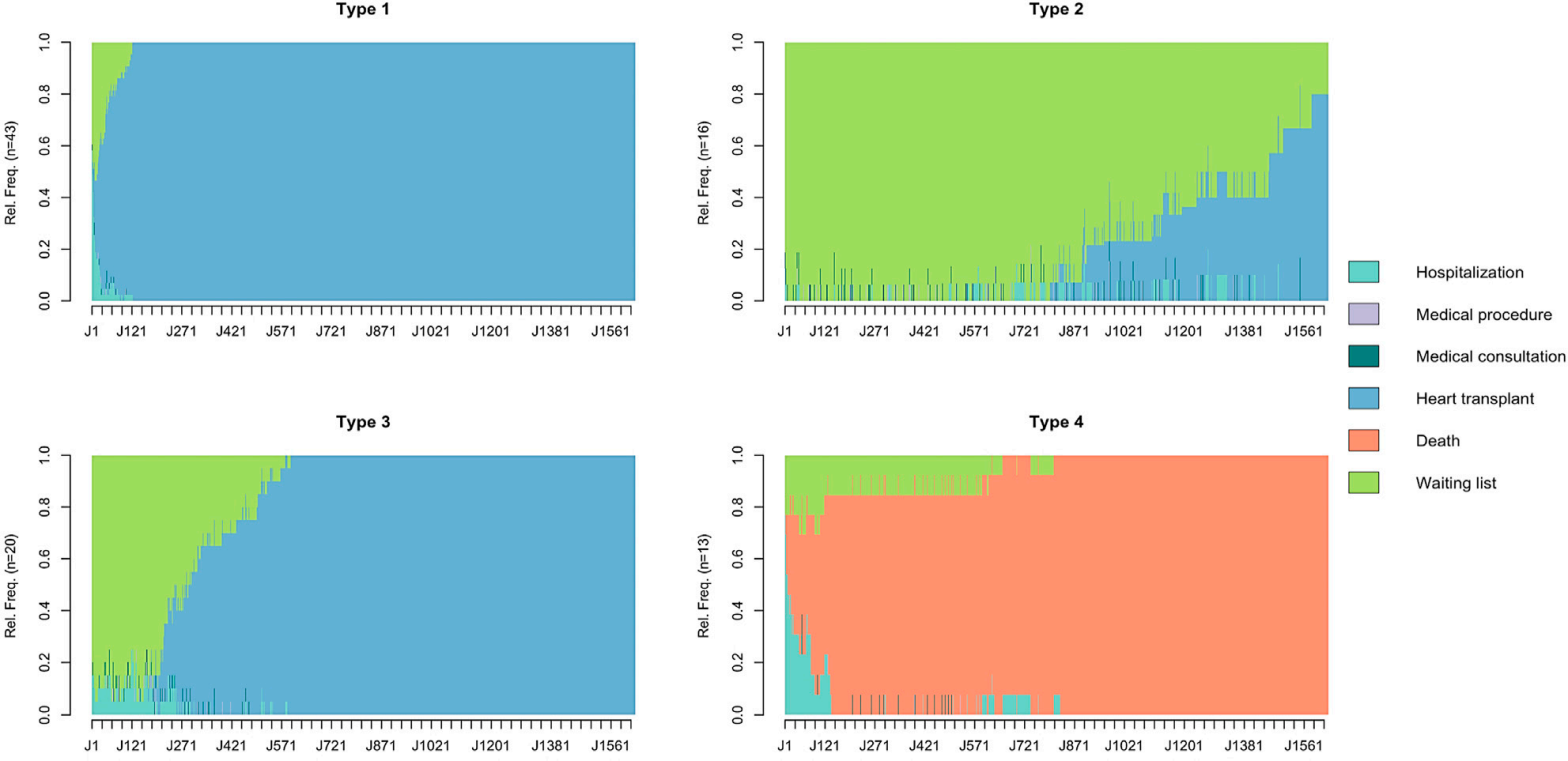
Chronograms of patient healthcare trajectories by cluster type. The X-axis represents the time from registration on the waiting list (time step = day). The Y-axis represents the relative frequency (Rel.Freq) of patients in the different states. Chronograms were obtained from a State Sequence Analysis with six states predefined: hospitalization, medical procedure, medical consultation, heart transplantation, death, waiting list. Optimal Matching was as the chosen dissimilarity measure method with an insertion/deletion cost of 1 and a substitution cost matrix estimated based on observed transition rates between states. Agglomerative hierarchical clustering using Ward’s criterion on the dissimilarity matrix was then performed to create homogeneous clusters of patients and optimal number of clusters was chosen using the inertia curve. Four homogeneous clusters of patients were identified (referred as “Types”).

After clustering, four homogeneous clusters of patients were identified based on the similarity of their healthcare trajectories (referred to as “Types” below). Chronograms are presented in Figure 1.

**FIGURE 2:**
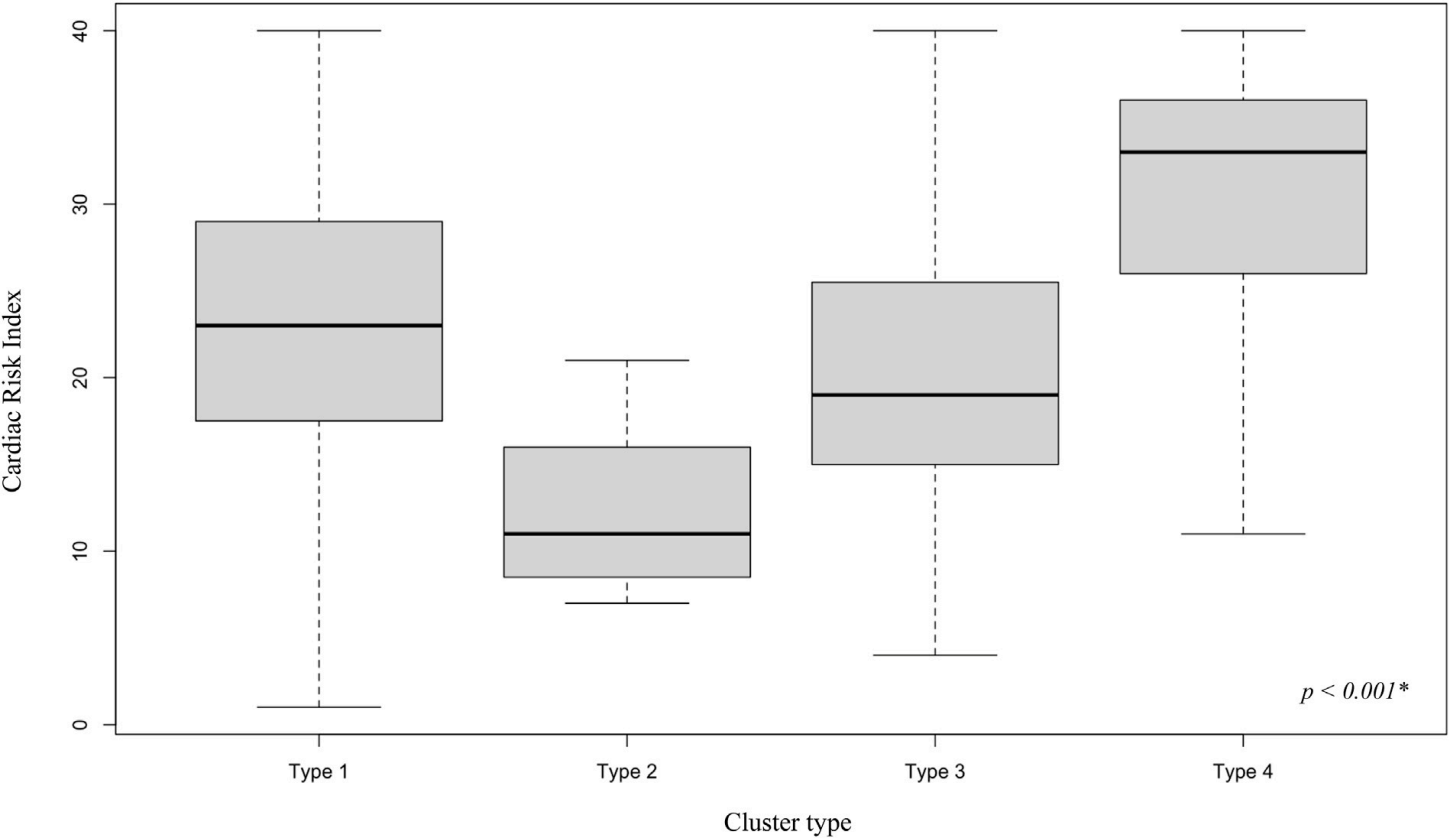
Cardiac Risk Index by cluster type. Distribution of the Cardiac Risk Index, a one-year waitlist mortality predictive score based on candidate characteristics, and part of the 2018 French heart allocation system, by cluster type. * Kruskall Wallis two-tailed test. A significance threshold of 5% was set.

Type 1 patients (n = 43, 47%) were predominantly NYHA Class III (51%), Type 2 patients (n = 16, 17%) NYHA Class II (50%) and NYHA Class III (44%), Type 3 patients (n = 20, 22%) NYHA Class III (70%) and Type 4 patients (n = 13, 14%) NYHA Class IV (62%). Type 4 patients were characterized by the highest median age (58 years). Temporary mechanical circulatory support was the leading support (46%) in Type 4 patients while durable mechanical circulatory support was the leading support (30%) in Type 3 patients. One patient (6%) received durable mechanical circulatory support among Type 2 patients. The distribution of the CRI according to the type of cluster is shown in Figure 2.

Results from the exploratory bivariate analyses identified sex, hypertrophic cardiomyopathy (as the indication for heart transplantation), NYHA Class, CRI, temporary mechanical support, durable mechanical support, and inotropic support as patient covariates associated with cluster membership (Table 1).

### Follow-Up and Access to Heart Transplantation

The median follow-up was 4 months (Q1–Q3 = 1–14). Two (2%) patients were lost to follow-up and considered non-transplanted (worst-case scenario). During the follow-up period, 67 (73%) patients underwent heart transplantation, 12 (13%) remained non-transplanted and 13 (14%) died. All Type 1 and Type 3 patients underwent heart transplantation while only 4 (25%) patients of Type 2 were transplanted. Patients dead during the follow-up were exclusively Type 4 patients. Median wait time from listing to transplantation was 2 months (1–8) overall, 1 month (0–2) for Type 1, 30 months (28–32) for Type 2 and 10 months (7–15) for Type 3. Type 4 patients died at a median of 3 months (0–4) after listing.

### Costs

The mean total cost for the entire patient trajectory was €21,324.05 [95% CI: €14,661.89–€31,314.91], mainly driven by hospitalization-related costs of €21,004.68 [95% CI: €14,392.35–€31,242.44]. The mean number of hospitalizations was 2.71 [95% CI: 1.99–4.76] (Table 2). Hospitalization for heart failure was the most common reason for admission, accounting for 27.7% (n = 69) of all admissions (Table 3). Costs varied significantly between Type 1 patients (€5,820.12 [95% CI: €3,823.34–€9,448.58]) and all patients, as well as between Types 2, 3 and 4 patients. Type 4 patients (€61,858.45 [95% CI: €32,130.42–€103,396.4]) had significantly different costs from all patients and from Type 1 patients. Type 3 patients had the highest mean number of hospitalizations with 5 admissions [95% CI: 2.35–14.25], whereas Type 1 patients had the lowest with 1 admission [95% CI: 0.74–1.35]. Type 4 patients had the highest mean cost for hospitalizations (€61,550.7 [95% CI: €32,392.09–€103,561]), 3 (23%) patients receiving a durable mechanical circulatory support during a hospitalization. Type 2 patients had the highest mean number of hospital medical consultations and procedures, 6.75 [95% CI: 4.25; 9.44] and 2.38 [95% CI: 1.38; 3.69] respectively. Average costs per year are presented in Table 4.

**TABLE 2:** Direct healthcare resource consumptions and costs, in euro price year 2023 from the health system perspective.

	All patients (n = 92)	Type 1 (n = 43)	Type 2 (n = 16)	Type 3 (n = 20)	Type 4 (n = 13)
Overall patient trajectory, Mean cost (€) [95% CI]	21,324.05 [14,661.89; 31,314.91]	5,820.12 [3,823.34; 9,448.58]	22,285.32 [11,254.33; 50,850.47]	27,541.11 [13,654.4; 55,149.85]	61,858.45 [32,130.42; 103,396.4]
All hospitalizations, mean [95% CI]	2.71 [1.99; 4.76]	1 [0.74; 1.35]	4.12 [2.62; 6.69]	5 [2.35; 14.25]	3.08 [1.69; 5.85]
Mean cost (€) [95% CI]	21,004.68 [14,392.35; 31,242.44]	5,572.9 [3,537.69; 9,209.7]	21,683.02 [10,588.7; 50,689.53]	27,285.41 [13,228.77; 53,641.06]	61,550.7 [32,392.09; 103,561]
Hospitalizations for heart failure, mean [95% CI]	0.75 [0.54; 1]	0.49 [0.3; 0.77]	0.62 [0.19; 1.6]	1.1 [0.55; 1.75]	1.23 [0.69; 1.77]
Mean cost (€) [95% CI]	10,812.55 [5,985.37; 18,915.73]	2,325.9 [1,260.87; 5,412.92]	3,171.05 [1,134.39; 9,308.26]	18,728.11 [5,558.03; 46,912.62]	36,110.92 [13,651.36; 74,642.67]
Hospital medical consultations, mean [95% CI]	3.46 [2.58; 4.54]	2.4 [1.44; 3.77]	6.75 [4.25; 9.44]	2.7 [1.25; 5.1]	4.08 [1.92; 8.92]
Mean cost (€) [95% CI]	190.11 [142.88; 250.49]	131.74 [79.3; 211.05]	371.25 [233.75; 522.5]	148.5 [66; 269.09]	224.23 [105.77; 477.12]
Hospital medical procedures, mean [95% CI]	1.36 [0.96; 1.84]	1.21 [0.7; 2.05]	2.38 [1.38; 3.69]	1.15 [0.5; 2.25]	0.92 [0.23; 2]
Mean cost (€) [95% CI]	129.26 [92.94; 176.3]	115.48 [65.31; 190.79]	231.05 [133.93; 374.01]	107.2 [49.31; 213.34]	83.52 [22.27; 181.88]

CI, confidence interval.

All costs were expressed in euros (€) at 2023 price year and adjusted for inflation based on the French National Institute of Statistics and Economic Studies (INSEE) Consumer Price Indices of the healthcare products and services.

Cost differences between groups were considered statistically significant if the bootstrapped 95% CIs did not overlap.

**TABLE 3:** Description of hospitalizations motives.

Hospitalization motives (n = 249 hospitalizations)	n (%)
Cardiac decompensation	69 (27.7%)
Cardiac examinations/assessments	43 (17.3%)
Infection related to the cardiovascular disease	19 (7.6%)
Arrhythmia	16 (6.4%)
Implantation/Follow-up/Complication of ICD	16 (6.4%)
Acute Kidney Injury	10 (4.0%)
Other cardiac-related hospitalizations	76 (30.5%)

ICD, Implantable Cardioverter Defibrillator.

**TABLE 4:** Average costs per year, in euro price year 2023 from the health system perspective.

Year of follow-up after waiting list inscription	All patients (n = 92)	Type 1 (n = 43)	Type 2 (n = 16)	Type 3 (n = 20)	Type 4 (n = 13)
First, n (%)	92 (100)	43 (100)	16 (100)	20 (100)	13 (100)
Mean cost (€) [95% CI]	16,616.3 [10,797.33; 25,770.64]	5,820.12 [3,823.34; 9,448.58]	3,665.76 [1,207; 12,045.37]	26,376.3 [12,323.29; 52,709.52]	53,941.49 [27,614.77; 90,905.34]
Second, n (%)	24 (26)	—	16 (100)	6 (30)	2 (15)
Mean cost (€) [95% CI]	6,818.52 [2,805.56; 14,706.02]	—	3,753.14 [839.23; 14,870.45]	4,408.46 [1,168.53; 12,021.89]	38,571.74 [27,029.34; 38,571.74]
Third, n (%)	15 (16)	—	14 (89)	—	1 (7)
Mean cost (€) [95% CI]	8,057.19 [3,741.51; 14,129.01]	—	6,924.94 [2,804.22; 12,868.08]	—	23,908.68^a^
Fourth, n (%)	9 (10)	—	9 (57)	—	—
Mean cost (€) [95% CI]	16,300.88 [1,076.51; 61,845.37]	—	16,300.88 [1,076.51; 61,845.37]	—	—
Fifth, n (%)	2 (2)	—	2 (14)	—	—
Mean cost (€) [95% CI]	951.18 [55; 951.18]	—	951.18 [55; 951.18]	—	—

CI, confidence interval.

^a^ Impossible to compute a confidence interval (n = 1).

All costs were expressed in euros (€) at 2023 price year and adjusted for inflation based on the French National Institute of Statistics and Economic Studies (INSEE) Consumer Price Indices of the healthcare products and services.

Cost differences between groups were considered statistically significant if the bootstrapped 95% CIs did not overlap.

## Discussion

To the best of our knowledge, this is the first economic evaluation of illness-related costs of patients with end-stage heart failure eligible for heart transplantation, using waiting list enrolment as the entry point. It is also the first study to characterize clusters of patients awaiting for heart transplantation based on their healthcare trajectories after listing.

The mean cost associated with managing these patients was €21,324.05, hospitalization being the main component. These results are consistent with a systematic review of cost-of-illness studies on heart failure published between 2004 and 2016, which found prevalence-based annual cost estimates ranging from $868 to $25,532 [19]. The review also found that hospitalization costs contributed significantly to total direct costs, from 44% to 96% [19]. However, few studies have focused on end-stage heart failure. Russo et al. estimated the mean cost of medical management of patients with advanced heart failure in the last 2 years of life in the United States, on the basis of the REMATCH trial (using date of death as reference point) to be $156,169, but this assessment was based on a health system significantly different from France, which may explain the higher costs, and included patients who were contraindicated to heart transplantation [20, 21]. Delgado et al. estimated costs for patients with symptomatic chronic heart failure in Spain, highlighting higher costs for patients with severe forms of heart failure including NYHA Class II (€3,789.30) and NYHA Class III-IV (€6,832.18) patients [22]. It was therefore of interest to use waiting list enrolment to define our end-stage heart failure population and assess its economic burden, as this population is usually difficult to characterize due to its inherent heterogeneity [23].

The state sequence analysis has also helped to understand patient pathways while waiting for transplantation, which is one of the objectives of the Ministerial Plan for Organ and Tissue Donation and Transplantation 2022–2026 in France [24]. Four clusters were identified. Type 1 patients had a low economic burden, as they survived until transplantation and were transplanted quickly (median 1 month). Despite high transplantation priority, Type 4 patients died before transplantation (median 3 months). The outcome of these patients, characterized by their critical condition, reflects the challenge of limited access to heart transplantation. Indeed, they are older (58 years), with 46% requiring ECMO and 77% dependent on inotropes, indicating greater severity. Their human leukocyte antigen (HLA) sensitization status would have been interesting but was not available. They also represented a major economic burden on the healthcare system with an average of 3 hospitalizations per patient. Additionally, 3 patients (23%) were bridged to heart transplantation on durable mechanical circulatory support after listing. These devices, funded separately from HRG-based fees and reimbursed in France at a price of €87,565, further contributed to the overall costs. Type 2 patients were not prioritized for heart transplantation. Consequently, they remained on the waiting list for an extended period, and only 4 (25%) patients underwent transplantation. Despite their initial milder condition, they still incurred significant healthcare costs due to deteriorating health, averaging 4.12 hospitalizations after listing. With 6 patients (30%) bridged to heart transplantation on durable support at enrolment, Type 3 patients underwent transplantation within a median of 10 months. However, they were heavy consumers of healthcare resources, averaging 5 hospitalizations and incurring higher costs compared to Type 2 patients over a significantly shorter period.

Heart transplantation remains the standard of care in selected, eligible patients, and is cost-effective [23]. This analysis further highlights the current issues related to its access, the economic consequences of organ shortage for healthcare systems, and the need to support strategies that can expand the donor’s pool [25–28]. Results from our COI study could therefore help inform decisions about health system resource allocation for this specific population and along the pathways identified [29, 30]. These results provide information on the economic burden of the disease, which could be reduced by health technologies designed to improve access to heart transplantation by expanding the donor pool, such as *ex vivo* perfusion systems [31, 32]. Indeed, our study showed that despite a priority status for transplantation, the average cost of patients who died before receiving a heart (i.e., Type 4 patients) was €61,550.7 [95% CI: €32,392.09–€103,561]. In comparison, the unitary purchase price of the consumables for one of these *ex vivo* perfusion systems (i.e., the TransMedics Organ Care System (OCS™) Heart (TransMedics; Andover, MA) is €54,000 including taxes (one consumable per procedure).

Therefore, we could hypothesize that the additional costs associated with the use of these expensive devices in routine in heart transplant centers, could be compensated by the reduction in the economic burden associated with the management of end-stage heart failure patients on the list, especially the most severe (i.e., Type 4). In addition, expanding the donor pool could lead to better health outcomes and health-related quality of life for these patients, which are of primary considerations within a cost-effectiveness analysis framework. These hypotheses need to be further investigated in a complete model-based cost effectiveness analysis. Here, we have provided real-world illness-related cost estimates in a French setting which could be further used for this economic evaluation and, more broadly for economic evaluations comparing treatment strategies for end-stage heart failure. Special emphasis should be placed on developing economic models based on real-world patient pathways [33].

Our study does have limitations. Data on changes in CRI during the time spent on the waiting list would have been interesting to capture changes in patient priority status, but the score was only reported at listing in the computerized medical records. Patients’ post-transplant prognosis and economic data according to their pre-transplant healthcare trajectory would also have been interesting. However, the primary objective of this study focused on the pre-transplant pathway, as economic data on these aspects are particularly scarce in the literature. In addition, a long follow-up period would have been required to collect this data. This retrospective cohort study was conducted in a single tertiary center and included a small number of patients. This limited the possibility to properly investigate associations between baseline patient characteristics (at the time of waiting list registration) and cluster membership using multivariate statistical modelling. This model could be of interest for predicting future healthcare trajectories and resource use based on patient characteristics at registration on the waiting list. These health economic estimates could be considered as complementary indicators for ranking candidates for heart allocation. Here, only exploratory bivariate analyses were conducted to identify which patient covariates may influence cluster type belonging (i.e., sex, hypertrophic cardiomyopathy as the indication for heart transplantation, NYHA Functional Classification, Cardiac Risk Index, temporary mechanical support, durable mechanical support and inotropic support). However, although these findings are exploratory and based on a small dataset, they may be of interest to clinicians managing these patients and involved in their care pathway. Furthermore, despite being single-centered, this study is a fairly good reflection of the French national situation in terms of access to heart transplantation over the same period, with one-year access at 76.7% [34]. The potential impact of the COVID-19 pandemic cannot be overlooked, as the number of heart transplants per year in France, according to data from the French Agency of Biomedicine, was 450 in 2018 and 425 in 2019, before decreasing to 370 in 2020, followed by 409 in 2021 and 411 in 2022 [35]. Of notice and in contrast with other solid organ transplant programs, heart transplant programs kept running during the COVID era and its access did not seem deeply affected by the outbreak. Finally, this economic evaluation was conducted from the healthcare system perspective and only focused on hospital care. A broader perspective may be of interest, especially when considering informal care, which may be an important cost component in end-stage heart failure [19]. However, this was not feasible here.

In conclusion, this study assessed the economic burden of patients waiting for heart transplantation and helped characterizing patients with higher healthcare resource utilization. It may provide insights for better informed decisions on the medical management of these patients, and help inform resource allocation along this pathway, particularly regarding strategies designed to expand the donor pool.

## Data Availability

The raw data supporting the conclusions of this article will be made available by the authors, without undue reservation.
