# Supplementary material for: Economic Burden and Healthcare Trajectories of Patients Awaiting Heart Transplantation in a French Tertiary Center
Source: Transpl Int. 2025 Mar 4;38:13703. doi: 10.3389/ti.2025.13703 (PMC11915721; doi:10.3389/ti.2025.13703)
Supplement: Supplementary file 1 [file Presentation1.pptx]

## Slide 1
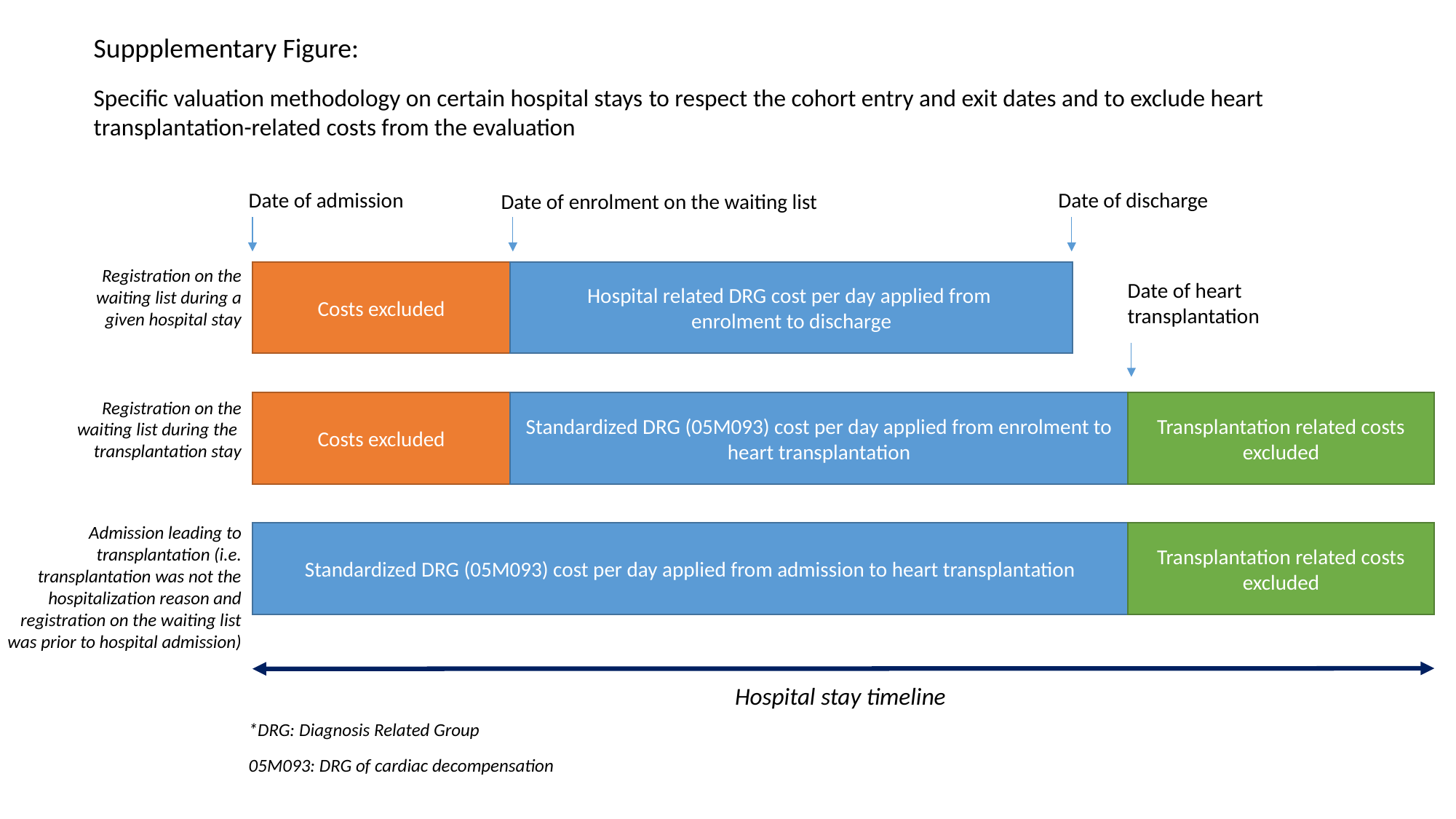

Suppplementary Figure:
Specific valuation methodology on certain hospital stays to respect the cohort entry and exit dates and to exclude heart transplantation-related costs from the evaluation
Date of discharge
Date of admission
Date of enrolment on the waiting list
Registration on the waiting list during a given hospital stay
Costs excluded
Hospital related DRG cost per day applied from
enrolment to discharge
Date of heart transplantation
Registration on the waiting list during the
transplantation stay
Costs excluded
Standardized DRG (05M093) cost per day applied from enrolment to heart transplantation
Transplantation related costs excluded
Admission leading to transplantation (i.e. transplantation was not the hospitalization reason and registration on the waiting list was prior to hospital admission)
Standardized DRG (05M093) cost per day applied from admission to heart transplantation
Transplantation related costs excluded
Hospital stay timeline
*DRG: Diagnosis Related Group
05M093: DRG of cardiac decompensation
